# Embryonic transcription is controlled by maternally defined chromatin state

**DOI:** 10.1038/ncomms10148

**Published:** 2015-12-18

**Authors:** Saartje Hontelez, Ila van Kruijsbergen, Georgios Georgiou, Simon J. van Heeringen, Ozren Bogdanovic, Ryan Lister, Gert Jan C. Veenstra

**Affiliations:** 1 Department of Molecular Developmental Biology, Radboud Institute for Molecular Life Sciences, Faculty of Science, Radboud University, PO Box 9101, 6500 HB Nijmegen, The Netherlands; 2 ARC Center of Excellence in Plant Energy Biology, The University of Western Australia, Perth, Western Australia 6009, Australia; 3 The Harry Perkins Institute of Medical Research, Perth, Western Australia 6009, Australia

## Abstract

Histone-modifying enzymes are required for cell identity and lineage commitment, however little is known about the regulatory origins of the epigenome during embryonic development. Here we generate a comprehensive set of epigenome reference maps, which we use to determine the extent to which maternal factors shape chromatin state in *Xenopus* embryos. Using α-amanitin to inhibit zygotic transcription, we find that the majority of H3K4me3- and H3K27me3-enriched regions form a maternally defined epigenetic regulatory space with an underlying logic of hypomethylated islands. This maternal regulatory space extends to a substantial proportion of neurula stage-activated promoters. In contrast, p300 recruitment to distal regulatory regions requires embryonic transcription at most loci. The results show that H3K4me3 and H3K27me3 are part of a regulatory space that exerts an extended maternal control well into post-gastrulation development, and highlight the combinatorial action of maternal and zygotic factors through proximal and distal regulatory sequences.

## Abstract (web summary)

Histone modifying enzymes are required for cell differentiation and lineage commitment during embryonic development. By a comprehensive set of epigenome reference maps of *Xenopus* embryos, the authors show that H3K4me3 and H3K27me3 exert an extended maternal control well into post-gastrulation development.

## Graphical abstract

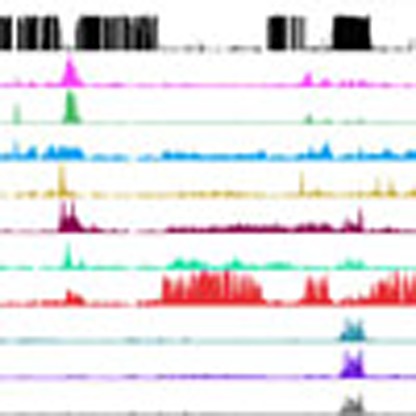

During early embryonic development cells differentiate, acquiring specific transcription and protein expression profiles. Histone modifications can control the activity of genes through regulatory elements in a cell-type-specific manner^1^,^2^,^3^,^4^. Recent advances have been made in the annotation of functional genomic elements of mammalian cells, *Drosophila* and *Caenorhabditis* through genome-wide profiling of chromatin marks^5^,^6^. Immediately after fertilization, the embryonic genome is transcriptionally silent, and zygotic genome activation (ZGA) occurs after a number of mitotic cycles^7^. In *Drosophila* and zebrafish (*Danio rerio*) ZGA starts after 8 and 9 mitotic cycles, respectively, in mammals transcription starts at the two-cell stage^8^,^9^, whereas in *Xenopus* this happens after the first 12 cleavages at the mid-blastula transition (MBT)^10^,^11^,^12^. Permissive H3K4me3 and repressive H3K27me3 histone modifications emerge during blastula and gastrula stages^13^,^14^,^15^,^16^. To date, little is known about the origin and specification of the epigenome in embryonic development of vertebrates, which is essential for understanding physiological cell lineage commitment and differentiation.

To explore the developmental origins of epigenetic regulation we have generated epigenome reference maps during early development of *Xenopus tropicalis* embryos and assessed the need for embryonic transcription in their acquisition. We find a hierarchical appearance of histone modifications, with a priority for promoter marks which are deposited hours before transcription activation on regions with hypomethylated DNA. Surprisingly, the promoter H3K4me3 and the Polycomb H3K27me3 modifications are largely maternally defined (MaD), providing maternal epigenetic control of gene activation that extends well into neurula and tailbud stages. By contrast, p300 recruitment to distal regulatory elements is largely under the control of zygotic factors. Moreover, this maternal-proximal and zygotic-distal dichotomy of gene regulatory sequences also differentiates between early and late Wnt signalling target genes, suggesting that different levels of permissiveness are involved in temporal target gene selection.

## Results

### Progressive specification of chromatin state

**Figure 1:**
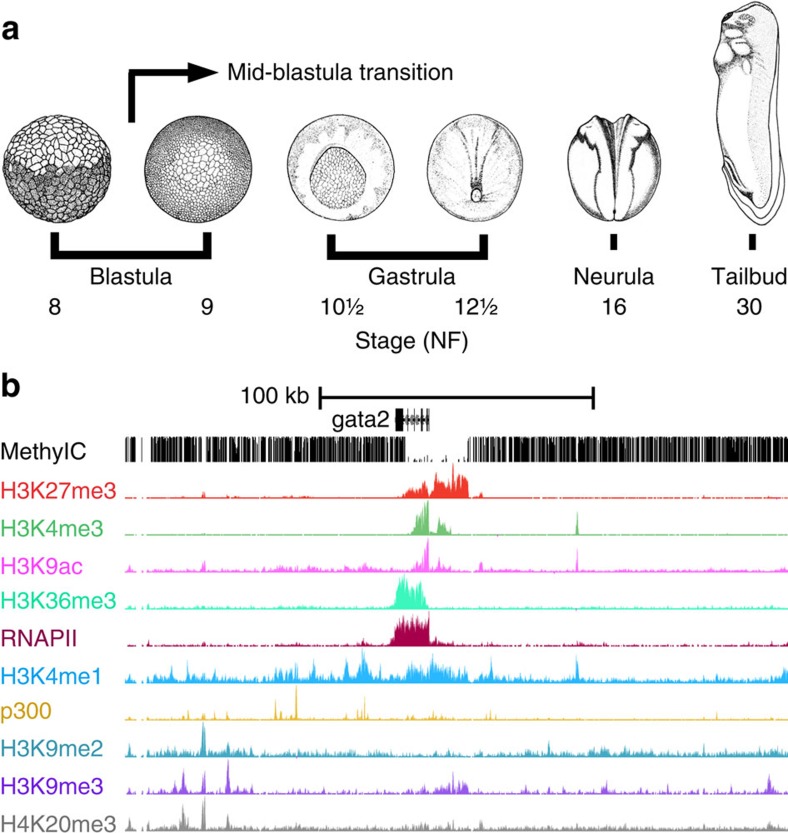
Reference epigenome maps of *Xenopus tropicalis* development. (**a**) Genome-wide profiles were generated for stages 8 and 9 (blastula, before and after MBT), 10.5 and 12.5 (gastrula), 16 (neurula) and 30 (tailbud). Adapted from Tan, M.H. *et al*. *Genome Res.* **23**, 201–216 (2013), under a Creative Commons License (Attribution-NonCommercial 3.0 Unported License), as described at http://creativecommons.org/licenses/by/3.0/. (**b**) *Gata2* locus with late gastrula (stage 10.5) methylC-seq, ChIP-seq enrichment of histone modifications, RNAPII and p300 (cf. Supplementary Figs 1 and 2).

**Figure 2:**
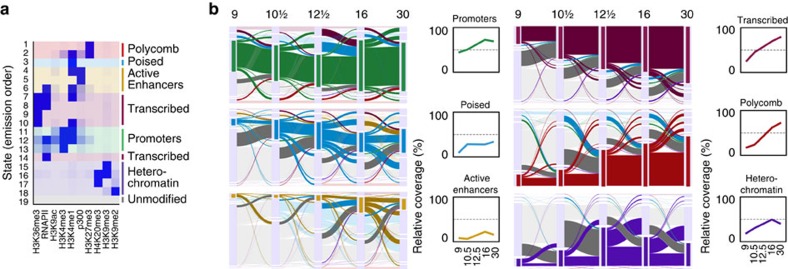
Chromatin state dynamics. (**a**) Emission states (same for all developmental stages) of the hidden Markov model, identifying the 19 most prevalent combinations of histone modifications and bound proteins. From top to bottom: Polycomb (red), Poised enhancers and promoters (blue), Active Enhancers (gold), Transcribed (dark magenta), Promoter (green), Heterochromatin (purple) and unmodified (grey). (**b**) Alluvial plots of chromatin state coverage during development. Each plot shows the transitions (to and from the highlighted group of chromatin states) across developmental stages (stages 9–30). The height represents the base pair coverage of the chromatin state relative to the modified genome. The ‘modified genome' has a chromatin state other than unmodified in any of the stages 9–30. From top to bottom left: promoters (green), poised (blue), p300-bound enhancers (gold). From top to bottom right: transcribed (dark magenta), Polycomb (red) and heterochromatin (purple). Line plots: Chromatin state coverage per stage as a percentage of the modified genome.

We have performed chromatin immunoprecipitation (ChIP) sequencing of eight histone modifications, RNA polymerase II (RNAPII) and the enhancer protein p300 at five stages of development: blastula (st. 9), gastrula (st. 10.5, 12.5), neurula (st. 16) and tailbud (st. 30). These experiments allow identification of enhancers (H3K4me1, p300)^17^,^18^,^19^,^20^, promoters (H3K4me3, H3K9ac)^14^,^21^,^22^,^23^, transcribed regions (H3K36me3, RNAPII)^22^ and repressed and heterochromatic domains (H3K27me3, H3K9me2, H3K9me3 and H4K20me3)^1^,^14^,^24^,^25^. In addition we generated pre-MBT (st. 8) maps for three histone modifications (H3K4me3, H3K9ac and H3K27me3) and single-base resolution DNA methylome maps using whole-genome bisulfite sequencing of blastula and gastrula (st. 9 and 10.5) embryos (Fig. 1; Supplementary Fig. 1). Our data set consists of 2.7 billion aligned sequence reads representing the most comprehensive set of epigenome reference maps of vertebrate embryos to date. Using a Hidden Markov Model approach^26^ we have identified 19 chromatin states based on co-occurring ChIP signals (Fig. 2a). This analysis identifies combinations of ChIP signals at specific genomic sequences without distinguishing between overlapping histone modifications that result from regional or cell-type specificity and co-occurrence in the same cells^14^. Seven main groups were recognized, namely (i) Polycomb (H3K27me3, deposited by Polycomb Repressive Complex 2 (PRC2)), (ii) poised enhancers, (iii) p300-bound enhancers, (iv) transcribed regions, (v) promoters, (vi) heterochromatin and (vii) unmodified regions (Fig. 2a; Supplementary Fig. 2). Alluvial plots of state coverage per stage show that all states increase in coverage during development, except for the unmodified state (Fig. 2b; Supplementary Fig. 2a). Unmodified regions decrease in coverage during development, however, even at tailbud stage 67% of the total epigenome remains naive for the modifications and bound proteins in our data set (Supplementary Fig. 2b). Promoter coverage remains relatively constant during development from blastula to tailbud stages, in contrast to the Polycomb state which increases in coverage during gastrulation. P300-bound enhancers are highly dynamic during development (Fig. 2b). Global enrichment levels of modified regions show similar dynamics, and reveal a priority for promoter marking at or before the blastula stage, followed by enhancer activation and heterochromatic repression during late blastula and gastrulation stages (Supplementary Fig. 3a,b). A detailed time course between fertilization and early gastrulation shows that both H3K4me3 and H3K9ac emerge hours before the start of embryonic transcription (Supplementary Fig. 3c). We and others have previously reported that H3K4me3 is acquired during blastula stages^14^. Indeed, H3K4me3 and H3K9ac levels increase strongly before the MBT, well before embryonic transcription starts. This however raises the question to what extent histone modifications are regulated by maternal or embryonic factors.

### Maternal and zygotic epigenetic regulation

**Figure 3:**
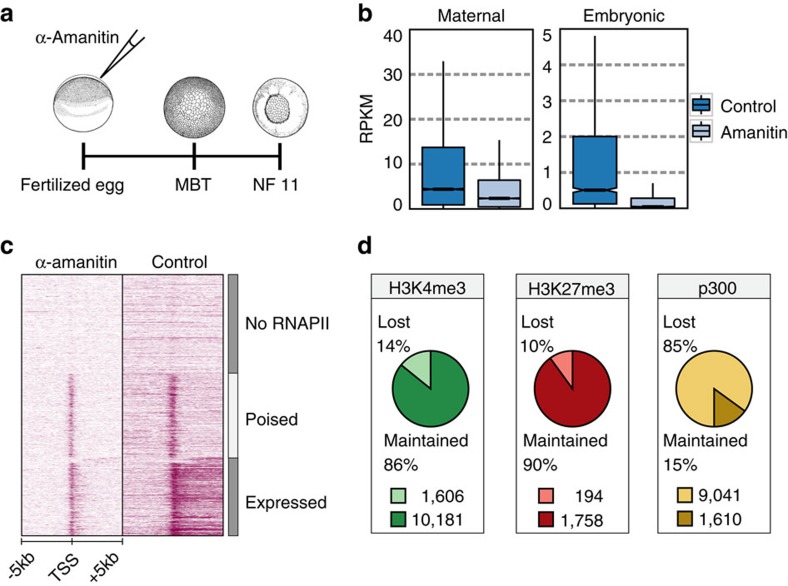
Developmental acquisition of chromatin states. (**a**) Inhibition of embryonic transcription with α-amanitin, adapted from Tan, M.H. *et al*. *Genome Res.* **23**, 201–216 (2013), under a Creative Commons License (Attribution-NonCommercial 3.0 Unported License), as described at http://creativecommons.org/licenses/by/3.0/. (**b**) RNAPII on the TSS of genes in control and α-amanitin-injected embryos (stage 11). (**c**) Box plots showing RNA expression levels (RPKM) of maternal and embryonic transcribed genes in control and α-amanitin-injected embryos (stage 11). Box: 25th (bottom), 50th (internal band), 75th (top) percentiles. Whiskers: 1.5 × interquartile range of the lower and upper quartiles, respectively. (**d**) ChIP-sequencing on chromatin of α-amanitin-injected and control embryos reveals maternal and zygotic origins of H3K4me3, H3K27me3 or p300 binding. Data from two biological replicates, see Supplementary fig. 4.

To determine the maternal and zygotic contributions to chromatin state, we used α-amanitin to block embryonic transcription (Fig. 3a). α-Amanitin blocks the translocation of RNA polymerase II (RNAPII) on DNA, thereby preventing transcript elongation^27^. It is therefore expected that injection of α-amanitin into embryos will stall RNAPII, immobilizing it on DNA after its recruitment to pre-initiation complexes. Indeed, both RNAPII elongation and embryonic transcription were effectively blocked in α-amanitin-injected embryos (Fig. 3b,c; Supplementary Fig. 4a). New transcription is necessary for gastrulation^11^,^28^,^29^, but α-amanitin-injected embryos survive to the equivalent of stage 11 control embryos. ChIP sequencing of replicates of α-amanitin-injected and control embryos (stage 11) revealed that the majority of H3K4me3 (86%) and H3K27me3 (90%) regions are consistently modified with these modifications independently of embryonic transcription (Fig. 3d; Supplementary Fig. 4b,c). This is especially surprising given the temporal hierarchy of H3K27me3 and H3K4me3, and the relatively late acquisition of H3K27me3 (Fig. 2b). By contrast, only 15% of the p300-bound regions recruit p300 independently of active transcription (Fig. 3d). This suggests that the promoter-permissive H3K4me3 mark and the Polycomb-repressive H3K27me3 mark are mostly controlled by maternal factors (maternally defined, MaD), whereas p300 binding to regulatory regions is largely zygotically defined (ZyD). Regions with MaD H3K4me3 and H3K27me3 acquire these modifications more robustly and also earlier during development compared with ZyD regions (Supplementary Fig. 4d). By contrast, ZyD p300-bound regions show more robust p300 recruitment during gastrulation compared with p300 MaD regions. These data show a pervasive maternal influence on the developmental acquisition of key histone modifications.

### DNA methylation logic of maternal control

**Figure 4:**
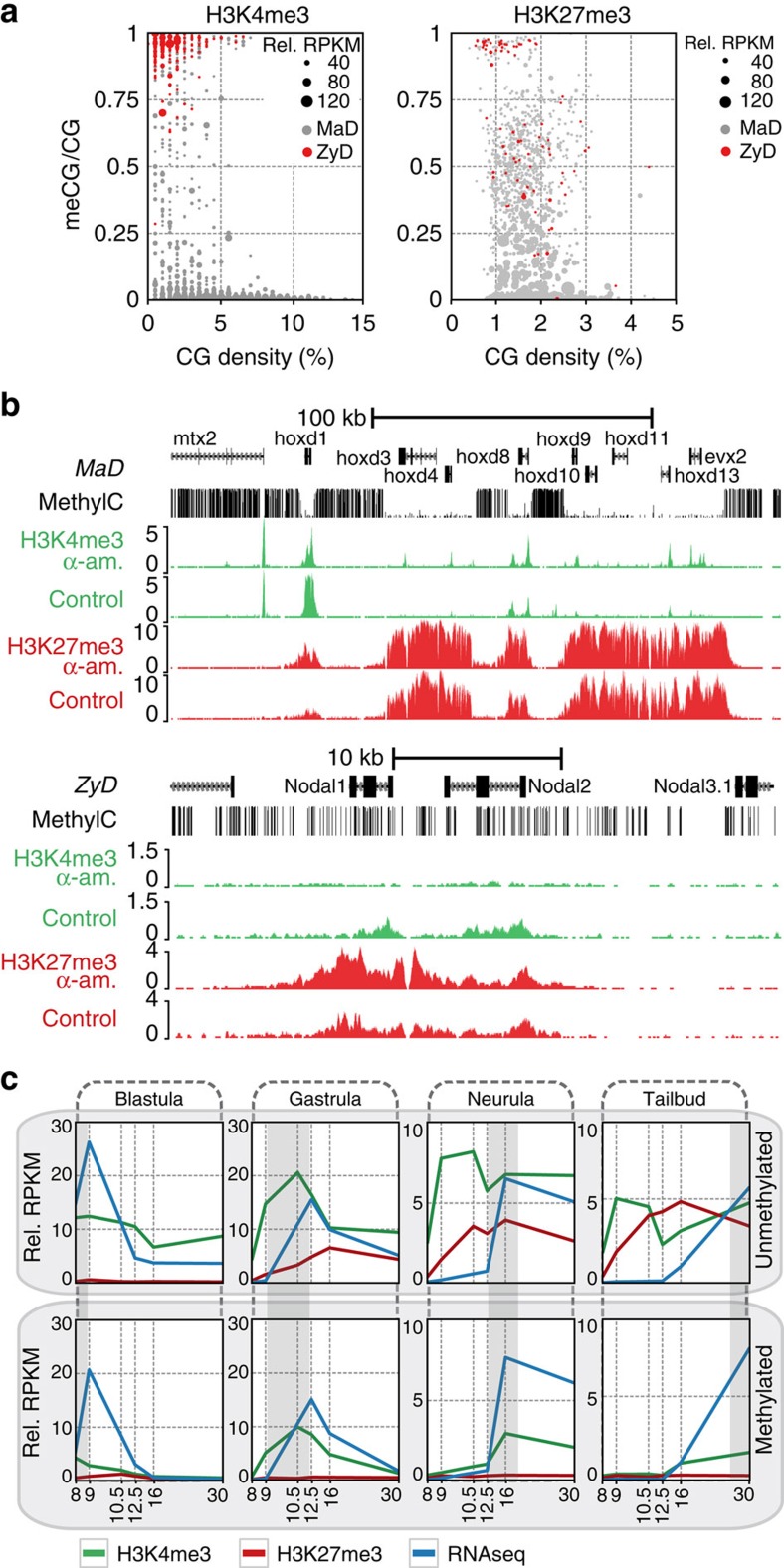
DNA methylation logic of maternally versus zygotically defined H3K4me3 and H3K27me3. (**a**) CpG density and methylation at stage 9 of promoters (H3K4me3: ±100 bp from TSS; H3K27me3: ±2.5 kb from TSS) that contain a zygotic defined (ZyD, lost in α-amanitin treated embryos, red) or maternal defined (MaD, maintained in α-amanitin treated embryos, grey) peak for H3K4me3 (left) or H3K27me3 (right) after inhibition of embryonic transcription. The size of the dot indicates the relative RPKM of the histone modification (background corrected). (**b**) *Hoxd* (MaD) and *nodal1, -2* (ZyD) loci with stage 9 methylC-seq, H3K4me3 and H3K27me3 in control and α-amanitin-injected embryos. (**c**) Developmental profiles of H3K4me3 and H3K27me3 (median background corrected RPKM) at genes without detectable maternal mRNA do correlate with activation for methylated promoters (lower panels) but not for hypomethylated CpG island promoters (upper panels).

Trimethylation of H3K4 and H3K27 has been associated with CpG density and a lack of DNA methylation. The Set1 and related MLL complexes are responsible for H3K4me3 (ref. 10). Set1 is recruited to hypomethylated CpG domains via the Cxxc1 protein (Cfp1)^30^,^31^,^32^. In the absence of H3K4me3, PRC2 binding to hypomethylated CpGs results in H3K27me3 and inhibition of gene activation^13^,^33^. Using our whole-genome bisulfite sequencing data we determined that MaD H3K4me3 promoters are predominantly hypomethylated (Fig. 4a; Supplementary Fig. 5a; Supplementary Data 1). Conversely, promoters decorated with ZyD H3K4me3 almost exclusively have highly methylated promoters. Demethylation of ZyD promoters was not detected, and methylation levels of MaD and ZyD regions were similar in stage 9 and stage 10.5 (Supplementary Fig. 5a,b). In addition, H3K4me3 often extends asymmetrically from promoters into gene bodies (+1–2 kb from transcription start site (TSS); Supplementary Fig. 5c), likely representing the second and third nucleosomes that are trimethylated via RNAPII-recruited Set1 in actively transcribed genes^34^. Concordantly, α-amanitin reduces H3K4me3 at downstream positions. Interestingly, we also find poised enhancers that gain H3K4me3 in α-amanitin-injected embryos and which exhibit intermediate to high levels of DNA methylation (Supplementary Fig. 5d,e).

The majority of promoters with ZyD H3K27me3 shows intermediate to high levels of DNA methylation (Fig. 4a; Supplementary Fig. 5a; Supplementary Data 1). Some of the MaD H3K27me3 regions are methylated, but the highly enriched H3K27me3 domains (larger dots) are almost exclusively both maternally defined and hypomethylated. This is illustrated by the *hoxd* cluster which harbours a large hypomethylated domain with MaD H3K4me3 and H3K27me3 (Fig. 4b). There are also examples of reciprocal changes of H3K4 and H3K27 methylation, for example at the hypermethylated promoters of *nodal1* and *nodal2*.

ZyD p300-bound regions are generally hypermethylated, whereas MaD p300-bound regions show a variable degree of DNA methylation (Supplementary Fig. 5e). However, promoters that overlap with MaD p300 peaks are hypomethylated in 77% of the cases, whereas 96% of the promoters that are associated with ZyD p300 peaks are hypermethylated (Supplementary Fig. 5f), showing that p300-recruiting hypomethylated promoters tend to be under complete maternal control, for both H3K4 methylation and p300 recruitment.

To further explore the relationships between DNA methylation, histone modifications and developmental activation of transcription we determined correlations with different measures of gene activity such as RNA-seq and ChIP-seq of RNAPII and H3K36me3 (Supplementary Fig. 6). We find that H3K36me3 and RNAPII in gene bodies correlate well with each other but less with transcript levels (RNA-seq), presumably due to the effects of RNA stability. A much lower correlation was found between either measure of gene activity and the promoter marks H3K4me3 and H3K9ac, especially at early stages. In part this may be caused by time delays of transcriptional activation relative to acquisition of permissive histone modifications^14^,^15^. It raises the question to what extent a lack of DNA methylation at promoters, which is associated with MaD H3K4me3, uncouples promoter marking and transcriptional activation. Therefore, we grouped transcribed genes without detectable maternal messenger RNA^35^ based on the stage of maximum expression and DNA methylation (Fig. 4c). We find that developmentally activated promoters with hypomethylated CpG islands are trimethylated at H3K4 or H3K27 early on, irrespective of the time of transcriptional activation. By contrast, methylated promoters show a much closer relation between H3K4me3 and gene expression. Although H3K4me3 is known to stabilize the transcription initiation factor Taf3 (a subunit of TFIID) and can also interact with the chromatin remodeller Chd1 (refs 36, 37, 38), hypomethylated promoters gain H3K4me3 autonomously with their hypomethylated CpG island status, independent of embryonic transcription.

### ZyD p300-bound domains shape enhancer clusters

**Figure 5:**
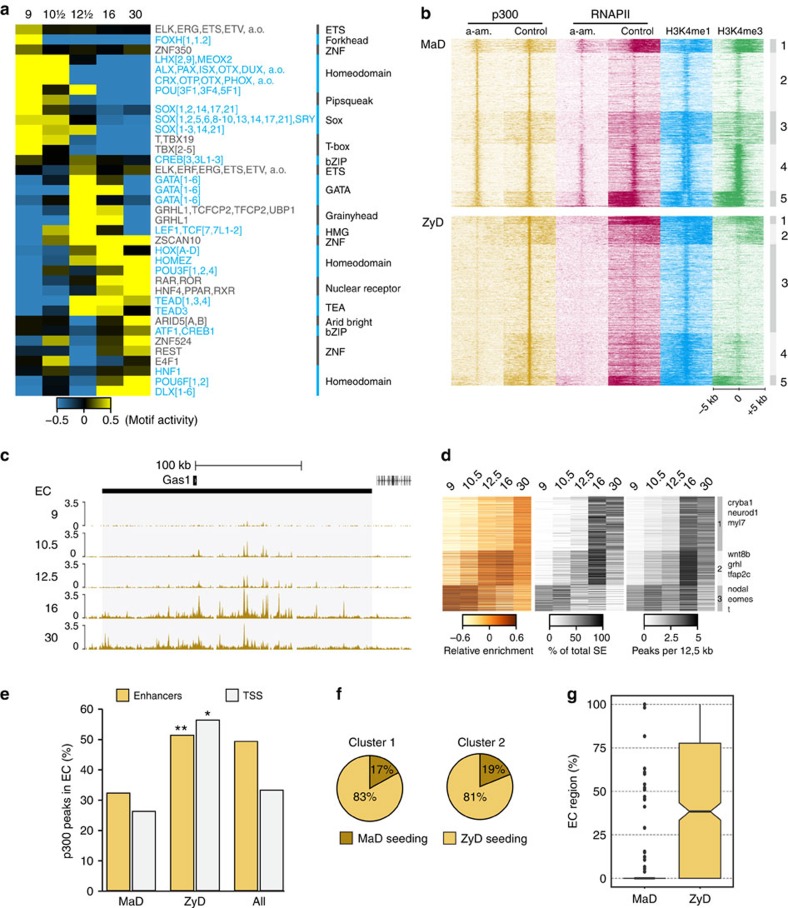
Zygotically controlled p300 recruitment shapes enhancer clusters (EC) domains. (**a**) Modelled transcription factor motif activity to p300 enrichment (see Methods). Activity reflects modelled contributions in p300 peak RPKM. (**b**) Heatmaps of MaD (upper panel) and ZyD (lower panel) p300 binding sites in α-amanitin treated and control embryos. (**c**) Developmental increase in genomic coverage of the *gas1* EC by acquisition of p300 binding at enhancers. (**d**) EC dynamics of p300 enrichment (left panel), percentage of total EC region identified in each stage based on stage-dependent p300 binding (middle panel) and number of p300 peaks (per 12.5 kb) in EC. (**e**) Percentage of zygotic defined (ZyD, lost in α-amanitin treated embryos) and maternal defined (MaD, maintained in α-amanitin treated embryos) p300 peaks that map to ECs. Asterisks indicate significance as more or less p300 peaks than expected by chance calculated using cumulative hypergeometric test: *P=6E−14; **P=5E−29 (**f**) Percentage of ECs that have a MaD or ZyD seeding peak at stage 9. (**g**) Box plot showing the percentage of the EC region that is defined by MaD or ZyD p300 peaks. Box: 25th (bottom), 50th (internal band), 75th (top) percentiles. Whiskers: 1.5 × interquartile range of the lower and upper quartiles, respectively. Outliers are indicated with black dots.

P300 can be recruited by transcription factors that bind to regulatory elements. We therefore modelled transcription factor motif contributions to p300 binding across multiple developmental stages (see Methods). The results predict specific transcription factors to recruit p300 in a stage-specific manner (Fig. 5a). Clustering of MaD and ZyD p300-bound regions with H3K4me3, H3K4me1 and RNAPII data revealed that ZyD p300 is recruited to distal regulatory sequences that lose both p300 and RNAPII binding in the presence of α-amanitin, whereas MaD p300 binding mostly includes promoter-proximal regions that are H3K4me3-decorated and recruit RNAPII in the presence of α-amanitin but without elongating (Fig. 5b). Indeed, MaD p300 regions are enriched for promoter-related motifs (Supplementary Fig. 7). Although some ZyD p300-bound regions overlap with annotated transcription start sites (Supplementary Fig. 5f), most of these sequences are decorated with H3K4me1 in the absence of H3K4me3, suggesting they correspond to distal regulatory sequences (Fig. 5b). Both MaD- and ZyD p300-bound regulatory regions recruit embryonically regulated transcription factors such as Otx2, Gsc, Smad2/3, Foxh1, T (Xbra), Vegt and Eomes (Supplementary Fig. 8)^39^,^40^,^41^, suggesting that multiple transcription factors contribute to p300 recruitment.

Large enhancer clusters (ECs) are thought to improve the stability of enhancer–promoter interactions, are associated with genes coding for developmental regulators, and have been implicated in cell differentiation^42^,^43^,^44^. During development the cluster size of p300-bound enhancers grows dynamically by p300 seeding of individual enhancers (Fig. 5c,d, see Methods). Histone modifications and transcript levels of EC-associated genes are developmental stage specific, confirming the association of ECs with developmental genes (Supplementary Fig. 9; Supplementary Data 2). Analysis of the percentage of the total EC regions identified in each stage show that most p300-bound ECs increase in genomic coverage during development by newly gained p300 binding at enhancers (EC clusters 1 and 2), whereas a group of early ECs (EC cluster 3) decrease in coverage as a result of the decreasing number of p300 peaks that contribute to the EC.

We next examined how MaD and ZyD p300-bound regions contribute to p300-bound ECs. Approximately 50% of all ZyD p300-bound enhancers are located in ECs at stage 11. Among MaD p300-bound enhancers this fraction is much reduced (Fig. 5e). Similarly, a much larger fraction of ZyD p300-bound promoters is found in ECs compared with MaD p300-bound promoters. Up to 20% of the developmental ECs that are seeded at stage 9 have a MaD p300 seeding site (Fig. 5f). However, very few ECs can be called based on MaD p300, showing that formation of p300-bound enhancer clusters requires embryonic transcription (Fig. 5g).

### Extended maternal epigenetic control

**Figure 6:**
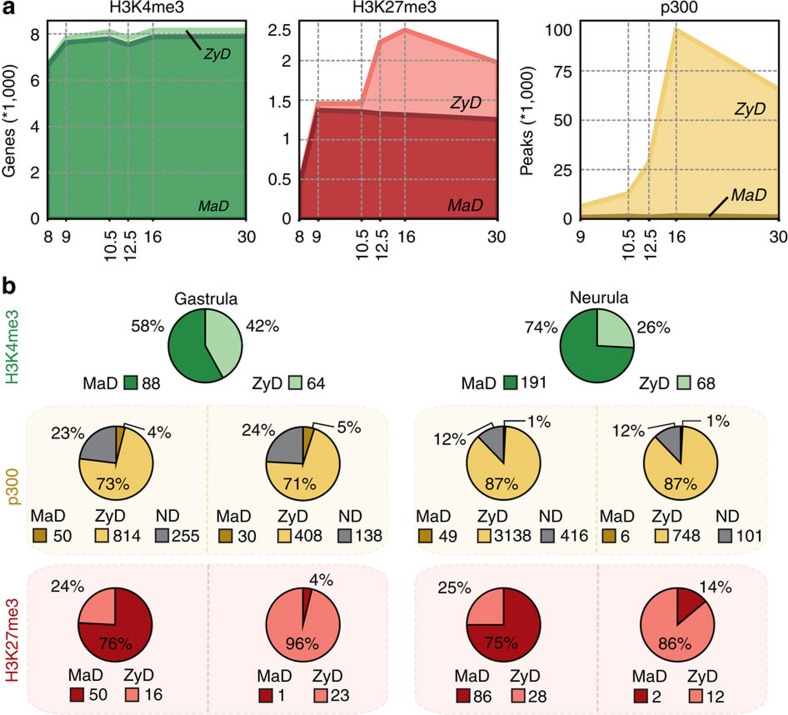
Maternal epigenetic control extends beyond gastrulation. Maternally defined (MaD) peaks emerge at or before stage 11 independent of embryonic transcription. Zygotically defined (ZyD) peaks appear before stage 11 and are lost in α-amanitin treated embryos, or emerge at or after stage 12. Not determined (ND) peaks are not consistently detected in replicates 1 and 2 and generally have low enrichment values. (**a**) Total number of genes with a MaD or ZyD peak in their promoter (H3K4me3 and H3K27me3), or total number of MaD and ZyD peaks per GREAT region (p300). ND peaks are not shown. (**b**) MaD and ZyD regulation of gastrula and neurula expressed genes. The pie charts show the number genes with a MaD or ZyD peak in their promoter (H3K4me3 and H3K27me3) or the number of MaD, ZyD and ND peaks per *cis*-regulatory region (p300). The H3K27me3 and p300 pie charts represent: Gastrula expressed genes with a MaD (far left) or ZyD (middle left) H3K4me3 peak; neurula expressed genes with a MaD (middle right) or ZyD (far right) H3K4me3 peak.

We next examined the extent to which the MaD epigenome is maintained during development. Genes were grouped based on MaD or ZyD trimethylation of H3K4 and H3K27 in the promoter (Supplementary Data 3, see Methods). For p300 we counted the total number of MaD and ZyD peaks in the *cis*-regulatory landscapes of genes (Fig. 6a). Remarkably, MaD H3K4me3-regulated genes represent the majority of all H3K4me3-enriched genes in both early and late developmental stages. Even at neurula and tailbud stages only a small fraction of the H3K4me3-decorated genes are ZyD. Similarly, maternal control of H3K27me3 also extends late into development, albeit to a smaller degree. After gastrulation, the number of MaD H3K27me3 regulated genes slightly decreases, whereas ZyD increases. However, also at neurula stage more than 50% of the Polycomb (PRC2)-regulated genes are under MaD H3K27me3 control. By contrast, p300 in *cis*-regulatory regions of genes is almost exclusively ZyD in all stages (Fig. 6a).

Many genes may maintain MaD H3K4me3 because they are constitutively expressed throughout development. We therefore analysed the regulation of genes that are exclusively embryonically transcribed. We find that 487 of 983 (49.5%) genes which are expressed between blastula and tailbud stages but not expressed in oocytes or before the MBT, feature a MaD H3K4me3 promoter (Supplementary Fig. 10a). Most of the MaD H3K4me3 genes that are modified by PRC2 exhibit MaD H3K27me3. When separating embryonic transcripts based on developmental activation, we find MaD H3K4me3 for 58% of the gastrula genes and up to 74% of the neurula expressed genes (Fig. 6b; Supplementary Fig. 10b). In most cases MaD H3K4me3-regulated genes also have MaD H3K27me3 control. This indicates an important role for the MaD epigenome in the regulation of embryonic transcripts.

**Figure 7:**
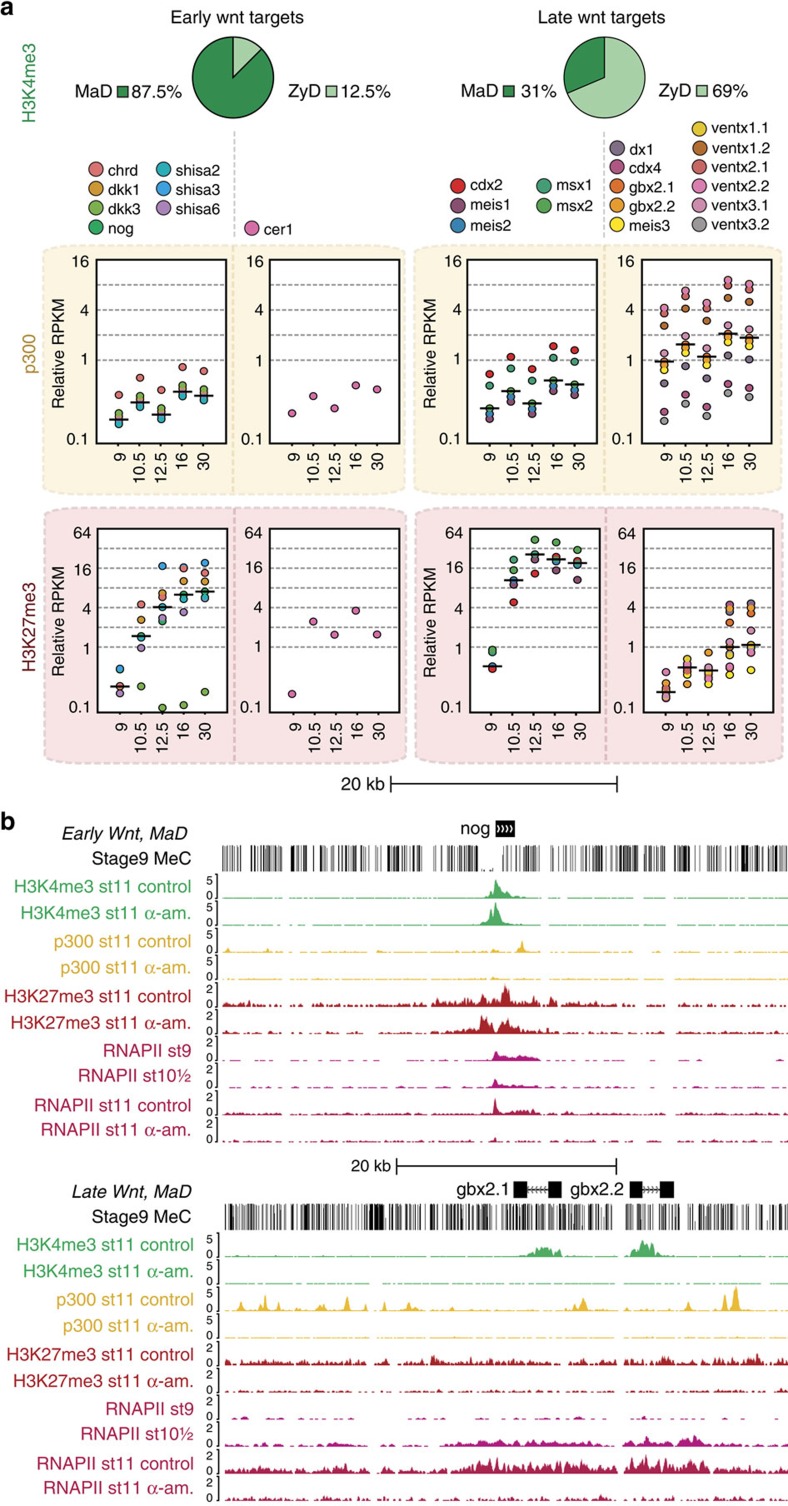
Maternal and zygotic regulatory space separates early and late Wnt target genes. (**a**) The number of genes with MaD or ZyD H3K4me3 (pie charts) and relative RPKM (dot plots, horizontal line: median) of p300 in *cis*-regulatory regions of genes and H3K27me3 on promoters (±2.5 kb from TSS) at different developmental stages that have maternally or zygotically defined H3K4me3 at the promoter. Early targets sia1 and sia2 are not included, these genes lose H3K4me3 after stage 9 and cannot be assigned to MaD or ZyD space based on our stage 11 α-amanitin data. H3K4me3 on these genes is acquired at stage 8, before embryonic transcription. (**b**) Browser views of the early Wnt target *nog (noggin)* and the late Wnt targets *gbx2.1* and *gbx2.2* with ChIP-seq enrichment of H3K4me3, p300 and RNAPII on control and α-amanitin-injected embryos and RNAPII on stages 9 and 10.5.

**Figure 8:**
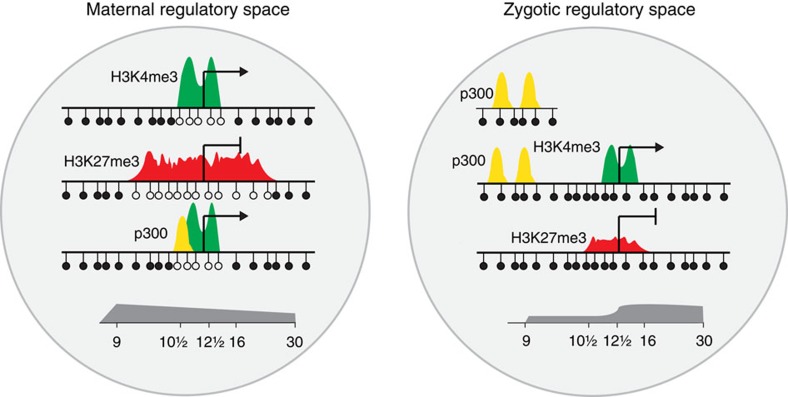
Model of maternal and zygotic regulatory space. This shows the segregation of maternal regulatory space, which contains hypomethylated promoters that are mainly controlled by maternal factors, and zygotic regulatory space, which includes methylated promoters and enhancers that are under zygotic control. Most p300-bound enhancers are in zygotic space, however, they can regulate promoters in both maternal and zygotic space, crossing the regulatory space border. This may contribute to varying degrees of permissiveness to transcriptional activation. Maternal regulatory space extends well into neurula and tailbud stages and includes many embryonic genes which are activated at specific stages of development. Zygotic regulatory space requires zygotic transcription, is established from the mid-blastula stage onwards but increases in relative contribution during development.

To explore the distinctions between expression inside and outside the maternal regulatory space, we analysed Wnt signalling targets. Early Wnt/beta-catenin signalling serves to specify dorsal fates following fertilization, leading to organizer gene expression. This has been shown to depend on Prmt2-mediated promoter poising before the MBT^45^. Indeed, we find that seven of eight early Wnt/beta-catenin targets have a hypomethylated island promoter marked with MaD H3K4me3 (Fig. 7a; Supplementary Fig. 10c). Wnt signalling also plays an important role after the MBT, when it ventralises and patterns mesoderm. The majority of these later targets turn out to have a methylated promoter with ZyD H3K4me3. Notably, these ZyD H3K4me3 late Wnt targets are associated with high binding of p300 in their locus; many of the p300 binding events happen at distal regulatory regions. In contrast, MaD H3K4me3 Wnt targets have less p300 binding but are marked with H3K27me3 (Fig. 7a,b). These results illustrate the dichotomy in proximal and distal regulation that is associated with transcriptional activation of maternal and zygotic Wnt target genes, which is paradigmatic of the distinctive maternal and zygotic epigenetic programs that are orchestrated by DNA methylation and exert a long-lasting influence in development (Fig. 8).

## Discussion

The H3K4me3 modification poises promoters for transcription initiation by stabilizing Taf3/TFIID binding^36^,^37^. Promoter H3K4 methylation based on an underlying DNA methylation logic driven by maternal factors at the blastula stage sets the stage for a default programme of gene expression. Most constitutively expressed housekeeping genes are within this maternal regulatory space, as well as a subset of developmentally regulated genes. Remarkably, many late expressed genes have hypomethylated promoters and are already poised for activation by H3K4me3 during early blastula stages. H3K4me3 is not sufficient for gene transcription and additional embryonic factors are required for activation in many cases. Genes with MaD H3K4me3 generally have fewer p300-bound enhancers associated with them, suggesting they are regulated by promoter-proximal elements. This further underscores the permissive nature of this regulation, as opposed to zygotically regulated events at both promoters (H3K4me3) and enhancers (recruitment of p300). The H3K27me3 modification is gradually acquired between blastula and gastrula stages on spatially regulated genes, repressing lineage-specific genes in other lineages^13^,^14^. The acquisition of this modification in the absence of transcription indicates that it is uncoupled from the inductive events of the early embryo, suggesting a default maternal response to a lack of transcriptional activation. The results indicate that maternal factors set permissions and time-dependent constraints on a subset of genes with reduced CpG methylation at their promoter. These permissions and constraints are likely to channel embryonic cell fates into a limited number of directions by controlling hierarchical developmental progression by master regulators. Previously we observed that DNA methylation does not lead to transcriptional repression in early embryos, whereas it does in oocytes and late embryos^46^. The observations described here suggest a new role of DNA methylation in defining a maternal-embryonic programme of gene expression. In zebrafish, the maternal methylome is reprogrammed between fertilization and ZGA, to match the paternal methylome. This also occurs in maternal haploid fish, and appears to align with CG content^47^,^48^, suggesting an intrinsic maternal mechanism that sets the stage for the MaD epigenome.

Gene expression outside maternal regulatory space could be mediated by p300-associated enhancers, most of which require new transcription for recruitment of p300. Promoter and enhancer activation in the ZyD regulatory space likely involves binding of specific factors. Indeed, we find that both MaD- and ZyD p300-bound regulatory regions recruit embryonically regulated transcription factors. Enhancers often contain binding sites for many different proteins, which can play different roles in opening up chromatin, recruitment of co-activators and establishing looping interactions with promoters. Future experiments will shed light on the maternal–zygotic hierarchy and the regulatory transitions underlying these events and the roles of maternal and zygotic pioneer factors. We find that ZyD p300-bound enhancers shape enhancer clusters. These form dense hubs of regulatory activity, and EC p300 binding is generally correlated with the expression of the associated genes. The work reported here suggests that recruitment of p300 to ‘seeding' enhancers precedes establishing cluster-wide activity of the local enhancer landscape. Future work will also need to address to which extent seeding causes relaxation and opening of the local chromatin and activity of neighbouring enhancers.

Key proteins of the molecular machinery involved in DNA methylation (Dnmt3a, Tet2), H3K4me3 (Mll1-4, Kdm5b/c), H3K27me3 (Ezh2, Eed, Kdm6a/b) and enhancer histone acetylation (p300) are not only highly conserved between species but also frequently mutated in cancer^49^,^50^,^51^. Moreover cancer-specific hypermethylated regions tend to correspond to Polycomb-regulated loci in embryonic stem cells and DNA methylation may restrict H3K27 methylation globally^52^,^53^. In addition, the sequence signatures of hypomethylated regions that acquire H3K4me3 or H3K27me3 are conserved between fish, frogs and humans^13^. These observations suggest that the molecular mechanisms that orchestrate the maternal and zygotic regulatory space are conserved. One key difference between mammals and non-mammalian vertebrates is the specification of extra-embryonic lineages between zygotic genome activation and the blastocyst stage in mammals^10^, so it is likely that the way this plays out for specific genes differs between species. In summary, our results provide an unprecedented view of the far reach of maternal factors in zygotic life through chromatin state. The dichotomy of maternal promoter-based and embryonic enhancer regulation demarcates an epigenetic maternal-to-zygotic transition that is maternal permissive to the expression of some embryonic genes and restrictive to others. This highlights the combinatorial interplay of maternal and zygotic factors through distinct mechanisms.

## Methods

### Animal procedures

*X. tropicalis* embryos were obtained by *in vitro* fertilization, dejellied in 3% cysteine and collected at the indicated stage. Fertilized eggs were injected with 2.3 nl of 2.67 ng μl^−1^ α-amanitin and developed until the control embryos reached mid-gastrulation (stage 11). Animal use was conducted under the DEC permission (Dutch Animal Experimentation Committee) RU-DEC 2012–116 and 2014–122 to G.J.C.V.

### ChIP sequencing and RNA sequencing

Chromatin for ChIP was prepared as previously described^54^,^55^, with minor modifications. Antibody was incubated with chromatin overnight, followed by incubation with Dynabeads Protein G for 1 h. The following antibodies were used: anti-H3K4me1 (Abcam ab8895, 1 μg per 15 embryo equivalents (Eeq)), anti-H3K4me3 (Abcam ab8580, 1 μg per 15 Eeq), anti-H3K9ac (Upstate/Millipore 06-942, 1 μg per 15 Eeq), anti-H3K36me3 (Abcam ab9050, 1 μg per 15 Eeq), anti-H3K27me3 (Upstate/Millipore 07-449, 1 μg per 15 Eeq), anti-H3K9me2 (Diagenode C15410060, 1 μg per 15 Eeq), anti-H3K9me3 (Abcam ab8898, 2 μg per 15 Eeq), anti-H4K20me3 (Abcam ab9053, 2 μg per 15 Eeq), anti-p300 (Santa Cruz sc-585, 1 μg per 15 Eeq) and anti-RNAPII (Diagenode C15200004, 1 μg per 15 Eeq). For all ChIP-seq samples of the epigenome reference maps and RNAPII ChIP-seq samples of the α-amanitin experiments three biological replicates of different chromatin isolations of 45 embryos were pooled. Two biological replicates for H3K4me3 (α-amanitin injected: 90 and 56 Eeq; control: 45 and 67 Eeq), H3K27me3 (α-amanitin injected: 90 and 180 Eeq; control: 45 and 202 Eeq) and p300 (α-amanitin injected: 112 and 56 Eeq; control: 112 and 67 Eeq) ChIP-seq samples of the α-amanitin experiments were generated. For RNA-seq samples of the α-amanitin experiments RNA from five embryos from one biological replicate was isolated and depleted of ribosomal RNA as previously described^35^. Samples were subjected to a qPCR quality check pre- and post preparation. Libraries were prepared with the Kapa Hyper Prep kit (Kapa Biosystems), and sequencing was done on the Illumina HiSeq2000 platform. Reads were mapped to the reference *X. tropicalis* genome JGI7.1, using STAR (RNA-seq) or BWA (ChIP-seq) allowing one mismatch.

### MethylC-seq

Genomic DNA from *Xenopus* embryos stages 9 and 10.5 was obtained as described before^56^. MethylC-seq library generation was performed as described previously^57^. Library amplification was performed with KAPA HiFi HotStart Uracil+ DNA polymerase (Kapa Biosystems, Woburn, MA, USA), using six cycles of amplification. Single-read MethylC-seq libraries were processed and aligned as described previously^58^.

### Quantitative PCR

PCR reactions were performed on a CFX96 Touch Real-Time PCR Detection System (BioRad) using iQ Custom SYBR Green Supermix (BioRad). We preformed RNA expression PCR (RT–qPCR (quantitative PCR)) and ChIP-qPCR for H3K4me3 and H3K9ac on promoters of *odc1*, *eef1a1o*, *rnf146*, *tor1a*, *zic1*, *cdc14b*, *eomes*, *xrcc1*, *drosha*, *gdf3*, *t*, *tbx2, fastkd3, gs17* (see Supplementary Methods for primer sequences). ChIP-qPCR enrichment over background was calculated using the average of 5 negative loci.

### Detection of enriched regions

We used MACS2 (ref. 59) with standard settings and a *q*-value of 0.05. Fragment size was determined using phantompeakqualtools^60^. Broad settings (--BROAD) were used for H3K4me1, H3K36me3, H3K27me3, H3K9me2, H3K9me3, H4K20me3 and RNAPII. Broad and narrow peaks were merged for H3K4me3. For H3K9ac narrow peaks were used. For p300 broad peaks were used in the ChomHMM analysis, narrow p300 peaks were used for super-enhancer and MaD versus ZyD analyses. All peaks were called relative to an input control track. Peaks that showed at least 75% overlap with 1 kb regions that have more than 65 input reads, and peaks that have a ChIP-seq RPKM higher than the 95 percentile of random background regions are excluded from further analysis. Only scaffolds 1–10 (the chromosome-sized scaffolds) were included in the analysis. Relative RPKM was calculated by dividing the ChIP-seq RPKM of the peaks by the ChIP-seq RPKM of the 95 percentile of random background regions.

We used MAnorm^61^ to determine differentially enriched regions in α-amanitin and control embryos. We used merged peak sets of replicate 1, replicate 2 and stage 10.5 to avoid bias caused by peak calling. Lost, gained and unchanged peaks per biological replicate were determined using the following parameters: lost peaks have *M*-values >1 and a −log base 10(*P* value) >5 (for H3K27me3) or 1.3 (for H3K4me3 and p300) and have a relative RPKM (background corrected) >1 in stage 11 control (no cut-off was used for st.11 control of H3K27me3 rep.1), stage 10.5 (H3K4me3 and p300) or stage 12 (H3K27me3); *increased peaks* have M-values smaller than -1 and a -log base 10(*P* value) >5 (H3K27me3) or 1.3 (H3K4me3 and p300) and have a rel. RPKM greater than 1 in stage 11 α-amanitin, stage 10.5 (H3K4me3 and p300) or stage 12 (H3K27me3); unchanged peaks are neither gained nor lost and have a rel. RPKM >1 in stage 11 control (no cut-off was used for st.11 control of H3K27me3 rep.1), stage 11 α-amanitin, stage 10.5 (H3K4me3 and p300) or stage 12 (H3K27me3). Maintained peaks are peaks that are not lost and have a rel. RPKM >1 in stage 11 control (no cut-off was used for st.11 control of H3K27me3 rep.1), stage 11 α-amanitin, stage 10.5 (H3K4me3 and p300) or stage 12 (H3K27me3). Common lost, gained, unbiased and maintained peaks are present in both replicates. All other peaks are considered not defined (ND). Replicate-specific peaks were only used for Supplementary Fig. 4b, for all other figures the common peaks were used.

DNA methylation levels in Supplementary Fig. 4d was calculated using previously published Bio-CAP data^62^. Bio-CAP RPKM levels of stage 11–12 were calculated for H3K4me3, H3K27me3 and p300 peaks, and corrected for input values. For Fig. 4c genes were considered ‘hypomethylated' if the Bio-CAP/Input ratio on the promoter (±1 kb from TSS) was >1.

RNA expression analysis was performed as previously published^35^. Embryonic transcripts were separated based on the clustering of maximum expression levels per stage in Fig. 3d of Paranjpe *et al.*^35^ (cluster 1=blastula, cluster 5=gastrula, clusters 3 and 4=neurula, clusters 2 and 6=tailbud).

Enhancer clusters were called as previously described^43^. Enhancer Clusters are called per stage and merged to determine the total Enhancer Cluster region. Percentage of the EC region is calculated relative to the total Enhancer Cluster region.

### MaD and ZyD classification

MaD peaks emerge at or before stage 11 and are also acquired in α-amanitin treated embryos in both replicates. Zygotically defined (ZyD) peaks appear at or before stage 11 and are lost in α-amanitin treated embryos in both replicates, or emerge after stage 11. To classify MaD and ZyD H3K4me3 genes we ran MAnorm on promoters (±250 bp from TSS) only, using similar restrictions as described in ‘Detection of enriched regions'. MaD H3K4me3 genes have a maintained promoter in both replicates, ZyD H3K4me3 genes have a lost promoter H3K4me3 peak in both α-amanitin replicates, or a peak that emerges after stage 11. MaD H3K27me3 genes have at least one MaD peak in the vicinity of their promoter (±2.5 kb from TSS). ZyD H3K27me3 genes have at least one ZyD peak in their promoter and lack a MaD peak. ND peaks or genes do meet the criteria for neither MaD nor ZyD. For p300 the total number of ZyD and MaD peaks was counted in GREAT^63^ regions of genes.

### ChomHMM analysis

Chromatin states were discovered and characterized using ChromHMM v1.10 (ref. 26), an implementation of a hidden Markov model. As input we used the enriched regions from ten tracks (H3K27me3, H3K36me3, H3K4me1, H3K4me3, H3K9ac, H3K9me2, H3K9me3, H4K20me3, p300 and RNAPII) across five developmental stages. We trained and ran the model with a range of states, and determined the 19 emission states model as the optimal number of states that could sufficiently capture the biological variation in co-occurrence of chromatin marks. We subsequently classified the states into seven main groups based on the presence and absence of specific chromatin marks.

The segmentation files of the seven main groups per stage were binned in 200 base pairs intervals. An *m* × *n* matrix was created, where *m* corresponds to the 200 base pair intervals and *n* to the developmental stages (9–30). Each element *a*(*i,j*) represents the chromatin state of interval *i* at stage *j*. For each chromatin group occurrences were counted per stage *n*. The changes between stage *n* and *n*+1 were plotted using Sankey diagrams ( https://github.com/tamc/Sankey), a flow diagram closely related to alluvial diagrams.

### Motif analyses

For the prediction of motif contribution to p300 recruitment (Fig. 5a) we have implemented the ISMARA method developed by Balwierz *et al.*^64^ This method uses motif activity response analysis to determine the transcription factors that drive the observed changes in chromatin state across samples. As input we used the number of known motifs found per p300 binding site and the RPKM of the p300 peaks per developmental stage. The model infers the unknown motif activities from the equation in which the changes in signal levels are explained with the number of binding sites and the unknown motif activities. Motifs that showed a *z*-score activity that was >13 are shown in Fig. 5a. Enriched motifs (Supplementary Fig. 7) were detected with gimme diff, a tool from the GimmeMotifs package^65^. The vertebrate motifs used in this script were obtained from CISBP ( http://cisbp.ccbr.utoronto.ca/)^66^ and clustered using gimme cluster from GimmeMotifs. The motifs are available at http://dx.doi.org/10.6084/m9.figshare.1555851 (ref. 67).

### Generation of plots and heatmaps

All heatmaps were generated using fluff ( http://simonvh.github.com/fluff)^13^ or gplots ( http://cran.r-project.org/web/packages/gplots/index.html). For all heatmap clustering, the Euclidean distance metric was used. Other plots were generated using ggplot2 ( http://ggplot2.org/).

## Additional information

**Accession codes**: The data generated for this work have been deposited in NCBI's Gene Expression Omnibus and are accessible through GEO Series accession number GSE67974. Visualization tracks are available at the authors' web site ( http://www.ncmls.nl/gertjanveenstra).

**How to cite this article**: Hontelez, S. *et al.* Embryonic transcription is controlled by maternally defined chromatin state. *Nat. Commun.* 6:10148 doi: 10.1038/ncomms10148 (2015).

## Supplementary Material

Supplementary Information Supplementary Figures 1-10 and Supplementary Methods

Supplementary Data 1 Transcription start sites of genes that have a MaD or a ZyD trimethylation of H3K4 or H3K27 at stage 11.

Supplementary Data 2 Genes that are closest to ECs for each cluster of Figure 5d.

Supplementary Data 3 Transcription start sites of genes that have a MaD, ZyD or ND trimethylation of H3K4 or H3K27 at one or more of the five developmental stages (9-30).
