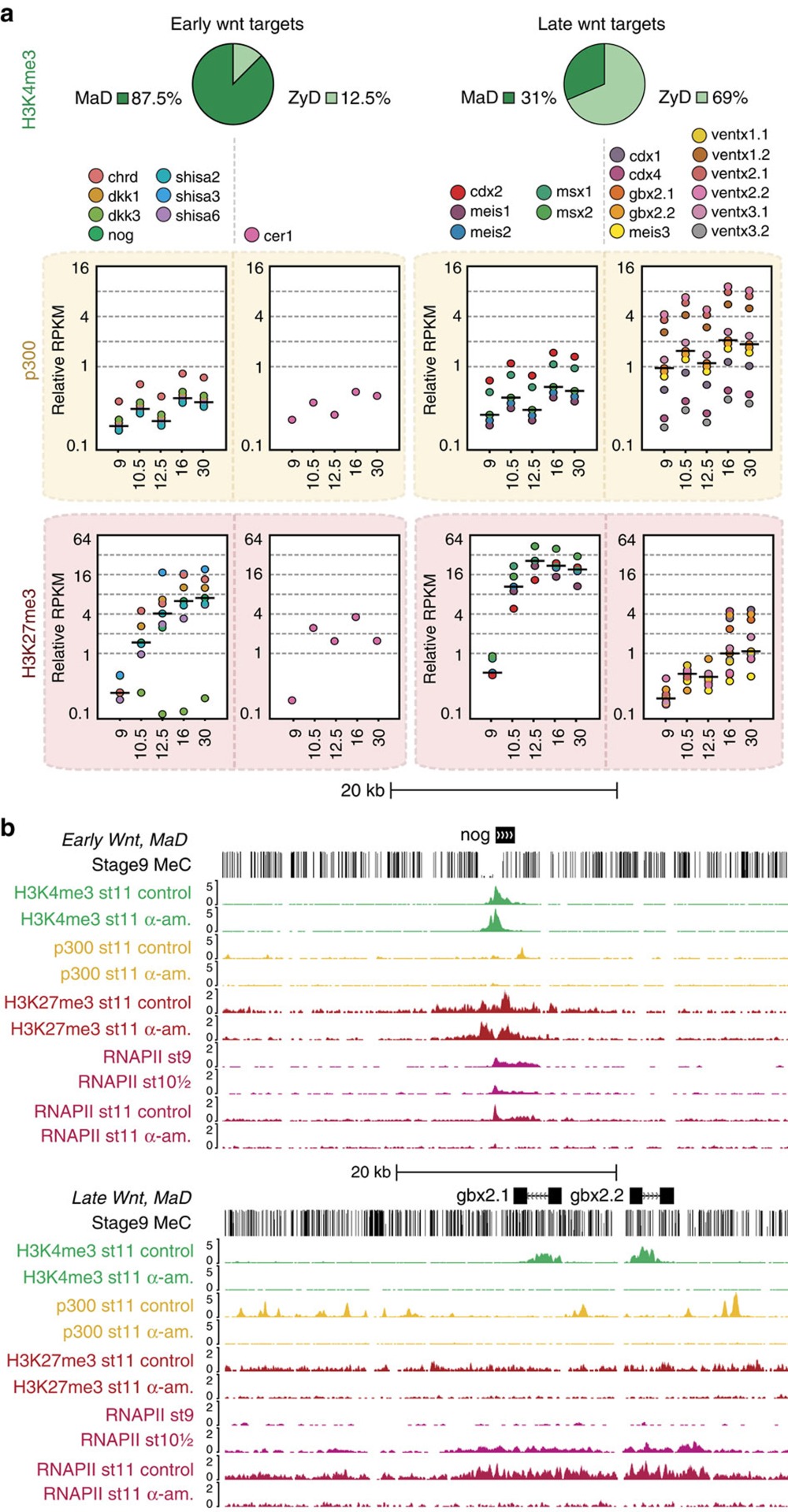# Erratum: Embryonic transcription is controlled by maternally defined chromatin state

**DOI:** 10.1038/ncomms12208

**Published:** 2016-07-05

**Authors:** Saartje Hontelez, Ila van Kruijsbergen, Georgios Georgiou, Simon J. van Heeringen, Ozren Bogdanovic, Ryan Lister, Gert Jan C. Veenstra

Nature Communications 6: Article number:10148 10.1038/ncomms10148 (2015); Published 12182015; Updated 07052016

This Article contains an error in Fig. 7 that was introduced during the production process. In panel a, the label ‘dx1' should read ‘cdx1'. The correct version of the figure appears below.

**Figure 7**