# Supplementary material for: Embryonic transcription is controlled by maternally defined chromatin state
Source: Nat Commun. 2015 Dec 18;6:10148. doi: 10.1038/ncomms10148 (PMC4703837; doi:10.1038/ncomms10148)
Supplement: Supplementary Information — Supplementary Figures 1-10 and Supplementary Methods [file ncomms10148-s1.pdf]

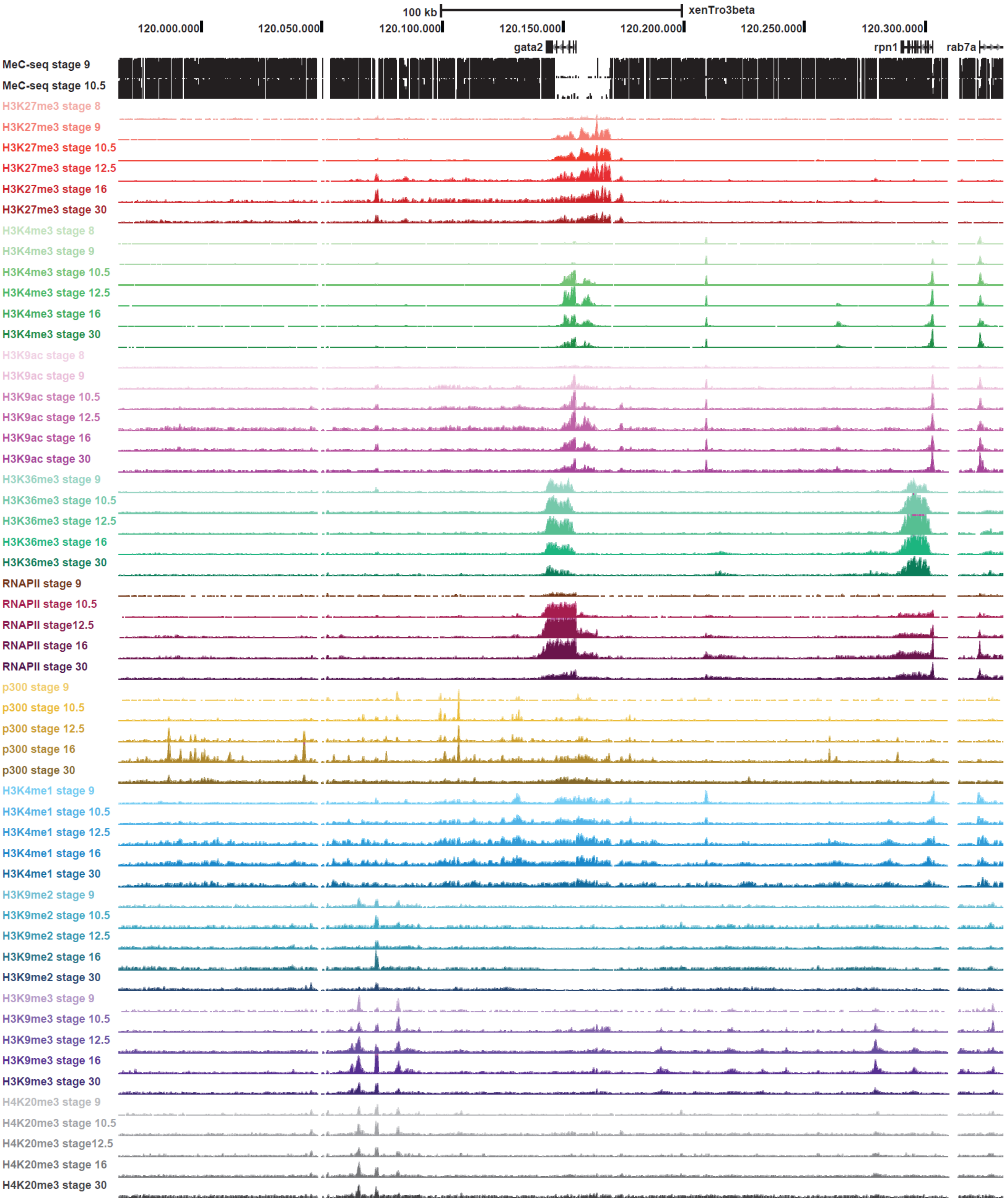

Supplementary Figure 1. *Gata2* locus. ChIP-seq enrichment of histone modifications, RNAPII and p300 for stages 9- 30. The heterochromatin tracks (H3K9me2, H3K9me3 and H4K20me3) are shown including non-unique sequence reads, identifying repetitive regions enriched for these modifications. ChIP-sequencing on stage 8 (blastula, pre-MBT) was done for histone modifications H3K4me3, H3K9ac and H3K27me3.

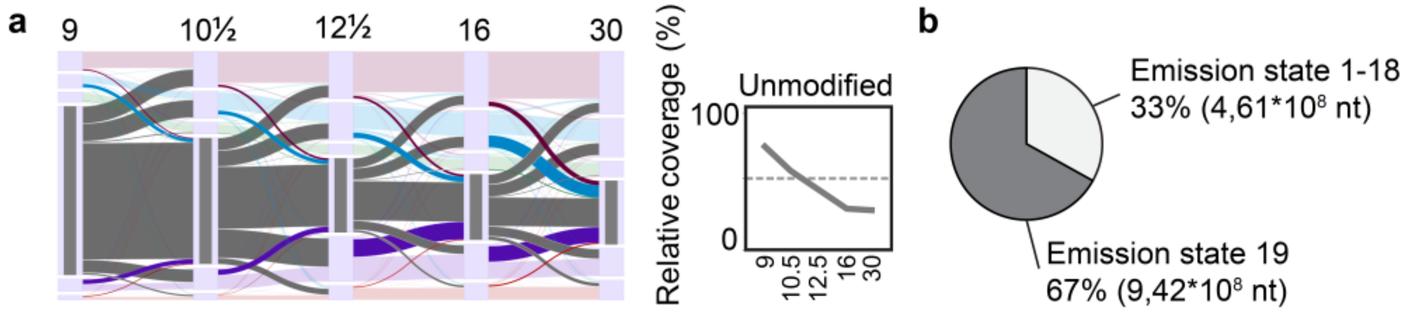

Supplementary Figure 2. Unmodified state coverage. (**a**) Alluvial plots of unmodified state (grey) coverage during development. The height represents the fraction of the modified genome that contributes to the same or a different chromatin state. The line plots shows coverage of the unmodified state per stage as a percentage of the sum of all regions that are state 1-18 at any stage. (**b**) Absolute nucleotide coverage of emission state 19 and states 1-18 at stage 30. It should be noted that 'unmodified' specifically refers to the examined histone modifications and that this state shows abundant DNA methylation.

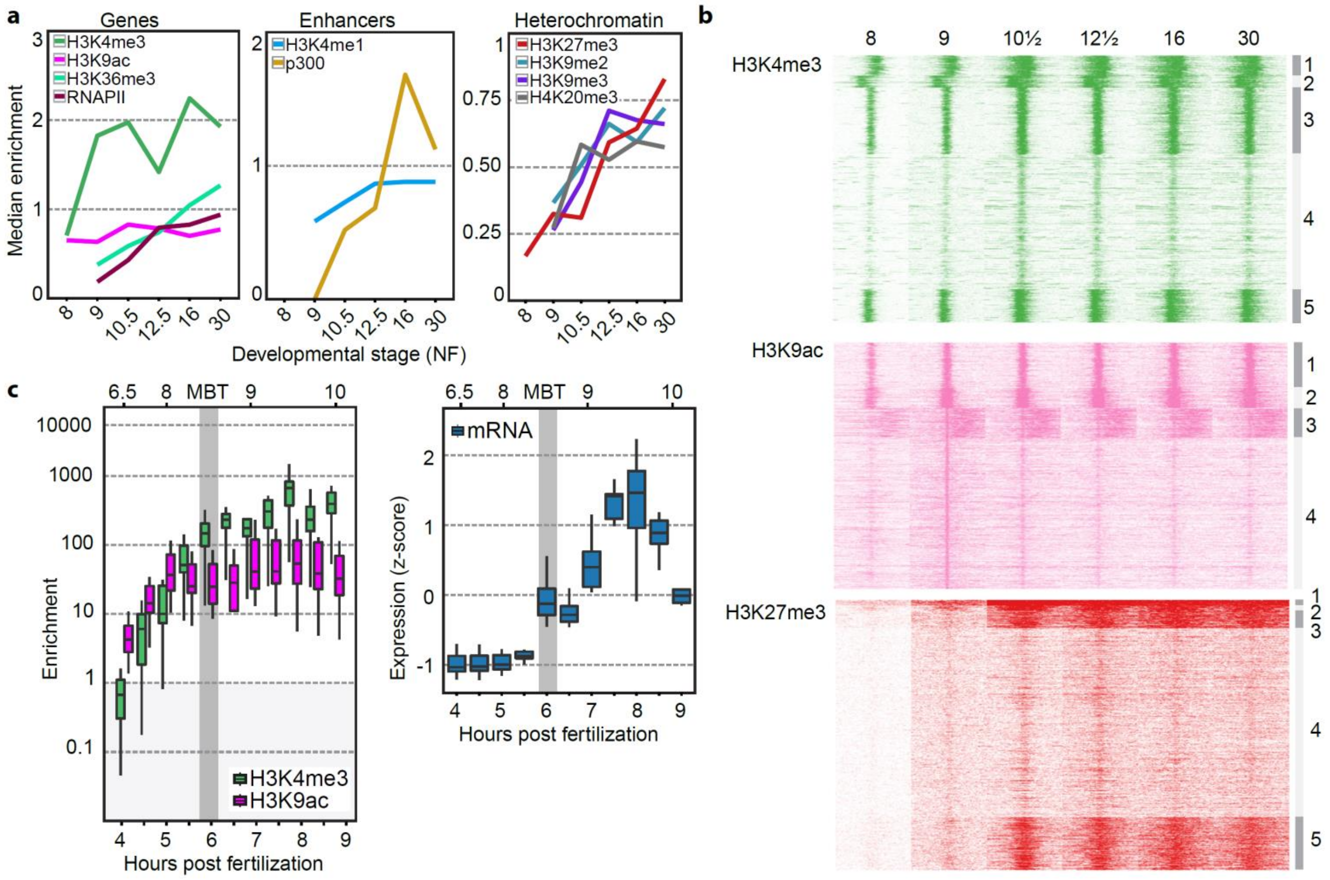

Supplementary Figure 3. Progressive specification of the epigenome. (**a**) Median enrichment of chromatin marks during development. (**b**) RPKM levels of H3K4me3, H3K9ac and H3K27me3 stage 8-30 on stage 9 peaks. Most stage 9 H3K4me3 and H3K9ac peaks show already significant enrichment at stage 8, whereas H3K27me3 markedly increases in late blastula and early gastrula embryos. (**c**) Detailed time series from 4 to 9 hours post fertilization (13 genes, average values of two biological replicates, see Methods). Left panel: Box plot of ChIP-qPCR for H3K9ac (pink) and H3K4me3 (green). Right panel: Box plot of RNA expression (RT-qPCR). Box: 25th (bottom), 50th (internal band), 75th (top) percentiles. Whiskers: 1.5 * interquartile range of the lower and upper quartiles, respectively.

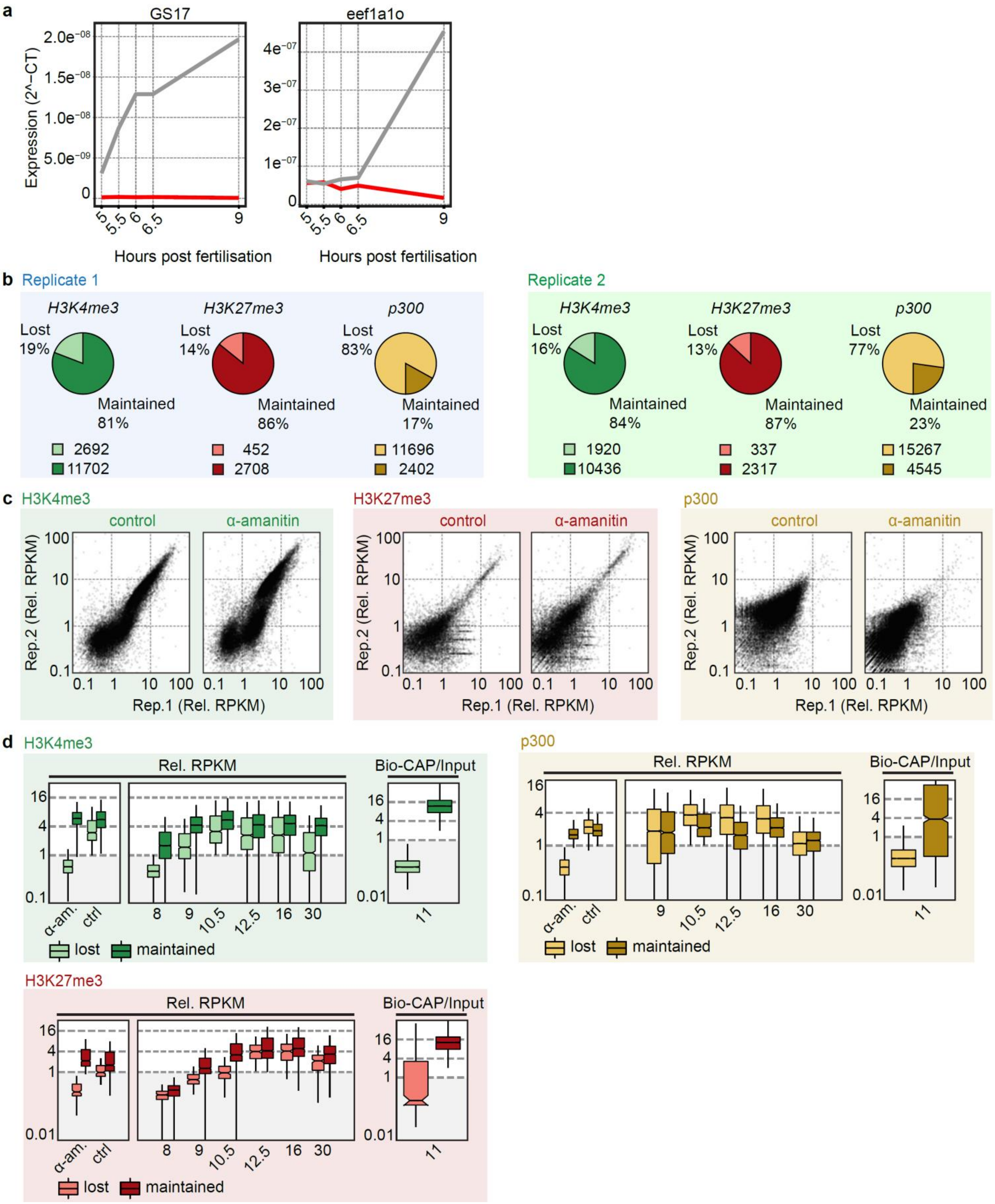

Supplementary Figure 4. Maternal and zygotic acquisition of chromatin state. (**a**) RNA expression (RT-qPCR) of *gs17* (embryonic transcript), *eef1a1o* (maternal transcript, induced at MBT) in α-amanitin and control embros. (**b**) Lost and Maintained peaks of H3K4me3, H3K27me3 and p300 in replicate 1 (left, blue background) and replicate 2 (right, green background). Pie charts representing percentage and numbers of lost and maintained peaks per replicate. (**c**) Scatter plots with relative RPKM (background corrected) of replicate 1 (x-axis) and replicate 2 (y-axis) on peaks that are lost or maintained in both experiments. (**d**) Left and middle panels show box plots of relative RPKM (background corrected) of regions with MaD or ZyD H3K4me3, H3K27me3 or p300-binding. Right panels show box plots of input corrected RPKM of previously profiled Bio-CAP data representing hypomethylated DNA domains [61]. MaD trimethylation of H3K4 and H3K27 is detected almost exclusively on Bio-CAP-enriched regions indicating clusters of hypomethylated CpGs. Box: 25th (bottom), 50th (internal band), 75th (top) percentiles. Whiskers: 1.5 * interquartile range of the lower and upper quartiles, respectively.

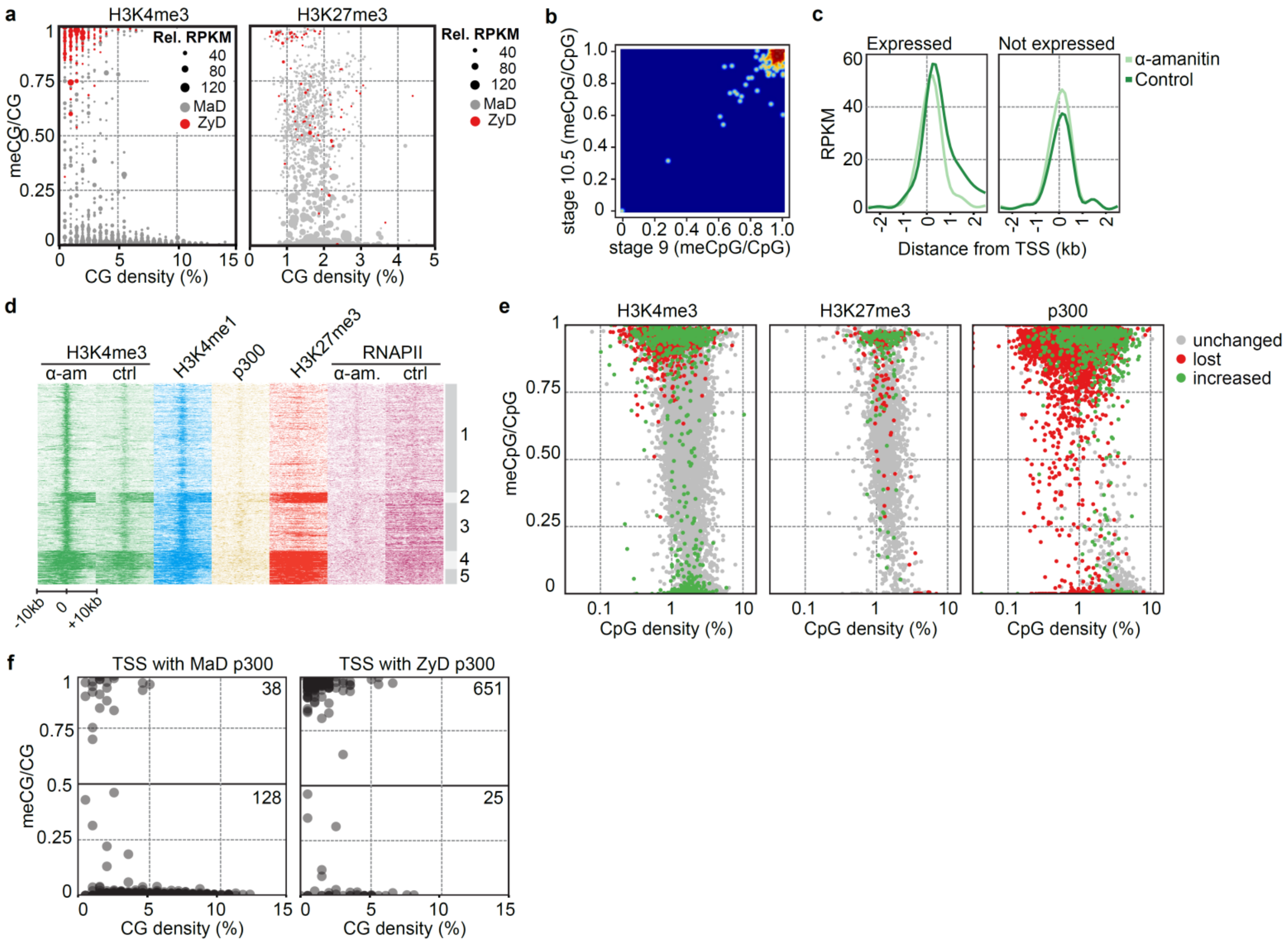

Supplementary Figure 5. Methylation logic for maternal and zygotic defined chromatin state. (**a**) CpG density and methylation at stage 10.5 of promoters (H3K4me3: ± 100 bp from TSS; H3K27me3: ± 2.5 kb from TSS) that contain a zygotic defined (ZyD, lost in α-amanitin treated embryos, red) or maternal defined (MaD, maintained in α-amanitin treated embryos, grey) peak for H3K4me3 (left) or H3K27me3 (right) after inhibition of embryonic transcription. The size of the dot indicates the relative RPKM (background corrected). (**b**) Density heatmap of DNA methylation stage 9 (x-axis) and stage 10.5 (y-axis) on ZyD promoters (± 100 bp from TSS). (**c**) Mean relative RPKM of stage 11 α-amanitin and control H3K4me3 on promoters of stage 10.5 expressed (left) and not expressed genes (right). (**d**) Heatmap representation of regions with increased H3K4me3 deposition in α-amanitin treated embryos. (**e**) CG density and methylation (stage 9) on lost, increased and unchanged H3K4me3 (left), H3K27me3 (middle) or p300 (right) peaks. For the purpose of simplicity, unchanged and increased peaks are collectively referred to as MaD in the rest of this article. (**f**) CG density and methylation on promoters (± 100 bp from TSS) that overlap with MaD (left) or ZyD (right) p300-bound peaks. The values in the middle and top corners indicate the number of promoters with meCG/CG ratio above or below 0.5.

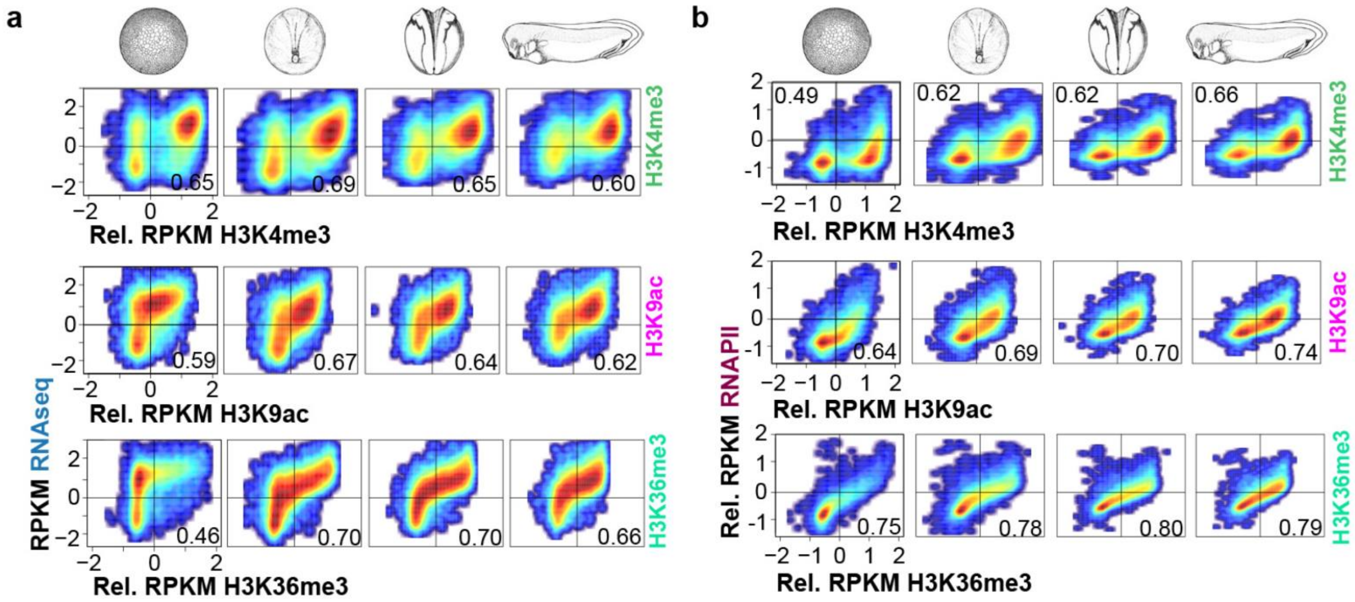

Supplementary Figure 6. Correlation of chromatin marks and transcription. Density correlation plots of relative RPKM (background corrected) for H3K4me3 and H3K9ac (± 1 kb from TSS) and H3K36me3 (genes bodies) with (**a**) RNAseq (exons) or (**b**) RNA polymerase II (gene bodies).

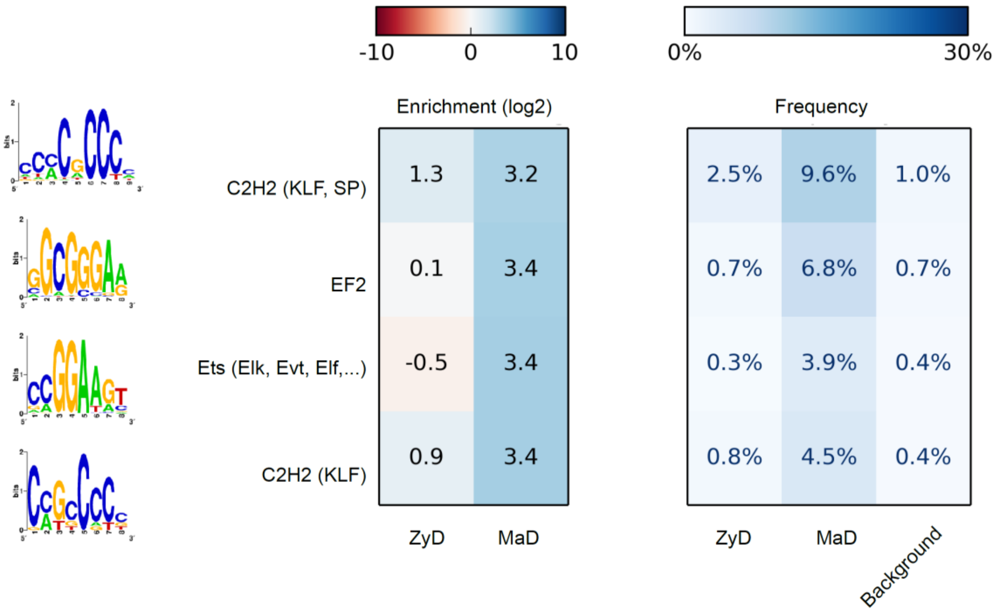

Supplementary Figure 7. MaD p300 regions are enriched for promoter related motif sequences. Motif enrichment and frequency in MaD and ZyD p300-bound regions.

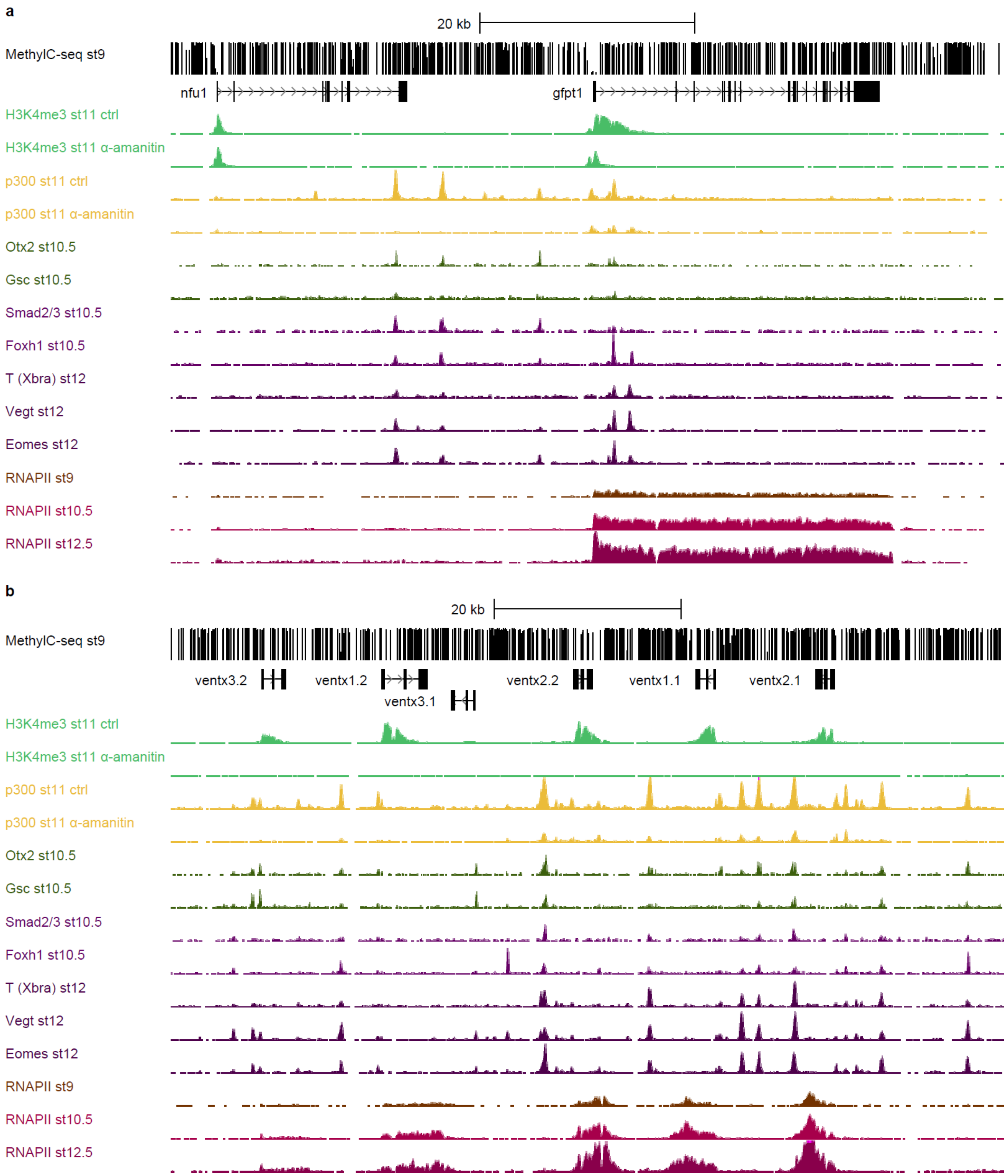

Supplementary Figure 8. MaD and ZyD p300 bound regions recruit embryonically regulated transcription factors. *Gfpt1* (**a**) and *ventx* (**b**) locus with stage 9 MethylC-seq and ChIP-seq enrichment of H3K4me3 and p300 on control and α-amanitin injected embryos, transcription factors Otx2, Gsc, Smad2/3, Foxh1, T (Xbra), Vegt, Eomes and RNAPII on stage 9 10.5 and 12.5.

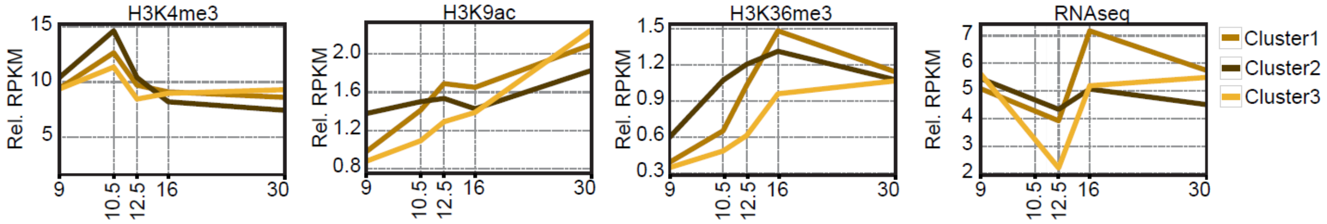

Supplementary Figure 9. Histone modifications and transcript levels of EC-associated genes. Median relative RPKM (background corrected) of H3K4me3 and H3K9ac (± 1 kb from TSS), H3K36me3 (gene bodies) and RNAseq (exons) for genes near ECs per heatmap cluster (Figure 3d).

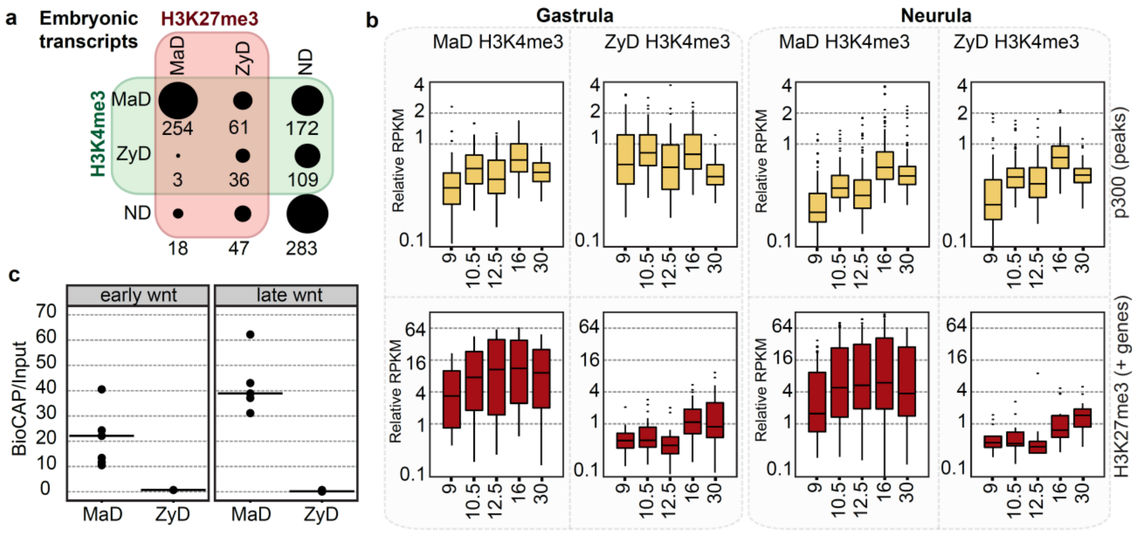

Supplementary Figure 10. Maternal and zygotic control of embryonic transcripts. Maternally defined (MaD) peaks emerge at or before stage 11 independent of embryonic transcription. Zygotically defined (ZyD) peaks appear before stage 11 and are lost in α-amanitin treated embryos, or emerge at or after stage 12. Not determined (ND) peaks are not detected in stage 11 control embryos. (**a**) Maternal and zygotic control of H3K4me3 and H3K27me3 on promoters of embryonic transcripts (total number of transcripts: 983). (**b**) Box plots of p300 RPKM (background corrected) in GREAT regions of genes, or H3K27me3 RPKM (background corrected) in promoters of genes with at least one H3K27me3 peak in their promoter (± 2.5 kb from TSS). Box: 25th (bottom), 50th (internal band), 75th (top) percentiles. Whiskers: 1.5 * interquartile range of the lower and upper quartiles, respectively. Outliers are indicated with black dots. (**c**) BioCAP enrichment (RPKM BioCAP/ RPKM Input) as a measure for hypomethylated DNA domains on the promoters (± 1 kb from TSS) of early and late Wnt target genes.

Supplementary Methods.

Primer sequences for RNA expression:

**Target**	**Forward (5’ → 3’)**	**Reverse (5’ → 3’)**
odc1	GTTTGTCCGAGGTGGTCTTT	CGGTGAAATAGAAGCCTGGT
eef1a1o	TGGAGGCTAGCACCAATATG	TGGAGGCTAGCACCAATATG
rnf146	TGTGCAAACCATCTTCTGCT	AAAAGATGCAGGGCTAGCAA
tor1a	ATCGAGCTGTGTTGGGTTTC	CAAAGTTCTTTCCGGTTCCA
zic1	TCCAACCACATCTGCTTCTG	TTGCAAACACTTTGCCACAT
cdc14b	AGAGGAGCAGCACAAAGGTC	TGACAGACTGGCACGGTAAC
eomes	ATCCCGGCCTCAGTACTCTT	GAGGAAGACCTGAGCTCGAA
xrcc1	AGCACGTGGTTTCCTGTAGC	TATGTGTCGGCTTTCAGCAG
drosha	GACACCGGTCTGAAAAGCAT	ACGGCTGCCATCTAGTCTTC
gdf3	CCTGCATGATGGAAGGTTTT	ATGGCTGCTACAGGTCCTTG
t	CAAGAATGGAAGGCGAATGT	GCGGTTGTTATCAGCAGTCA
tbx2	GAGAATGTTTCCCCCATTCA	CCGCAGCCACTATATCCATT
fastkd3	AAAATGGACATGCGGGATTA	TGCAGAAACGTTCGGTACAC
gs17	CGTGGGTCCTGGACTACTTT	GCAACTGCTTCAAAACCAGA

Primer sequences for ChIP-qpcr (H3K4me3 and H3K9ac):

**Target**	**Forward (5’ → 3’)**	**Reverse (5’ → 3’)**
odc1	GTTTGTCCGAGGTGGTCTTT	CCCCCGAAACACAATAAAAC
eef1a1o	CAACCCCCACGAACAAATAC	TTTAAATTCCAGCCGGTAGC
rnf146	GTACAGCGCTTTCCAAAAGG	GGTTTCGCCCCTCTCTTTAG
tor1a	GACAGCGATACCGAGAGTGA	AGCGCCTAGAGAGCTAATGG
zic1	CCATGTCAGCTCCTACTCCA	GGTTGCGAAAGAGAAAGTCC
cdc14b	TCTCTTACCGATTCCCCATC	TGCAAGGTGTAACCAGAGGA
eomes	CCTCTGGCTTAAATTCCACC	TAACCTTACCTGCCCTGCTT
xrcc1	TATTCCACAGCCGCAAATTA	AATAAATCTGGCAACCCTGG
drosha	TCCCCGTTTTAGATTCCAGT	CAAAGCATAACTGGGGAGGT
gdf3	CACCATTGTTTGAGGGCATA	CACCATTGTTTGAGGGCATA
t	TCGTCACCAAGAATGGAAGGT	GGGTAGAATAAGTGGAGAGACCCTG
tbx2	TGTCCAATGCACACACACAG	TGTCCAATGCACACACACAG
fastkd3	TACCCCTTACTCCAACAGGG	TAAATGCCAGTCTCCGTGAA
neg. locus 1	CATTCCCTACTGGGCTGGGT	CACACTGCTGGCCATCGTT
neg. locus 2	TGATTTTTTCACATATGCAGGCA	GTGTACATGCAGGTGGCAGTG
neg. locus 3	CATTAGTGCTAGCAGCCTTCC	TTCTCACCCCTTCATTCACC
neg. locus 4	GCCACTTGAAGCAACTTTCC	GACATGGTGAGATGCACTGG
neg. locus 5	CCTTGGCTCACTCTTTCAGG	TTGGAGGCAAGCGGTATAAG
